# Experience-Dependent *Egr1* Expression in the Hippocampus of Japanese Quail

**DOI:** 10.3389/fpsyg.2022.887790

**Published:** 2022-05-18

**Authors:** Chelsey C. Damphousse, Noam Miller, Diano F. Marrone

**Affiliations:** Department of Psychology, Wilfrid Laurier University, Waterloo, ON, Canada

**Keywords:** ZENK, avian, bird, place cell, navigation, cognitive maps

## Abstract

The hippocampal formation (HF) is a structure critical to navigation and many forms of memory. In mammals, the firing of place cells is widely regarded as the fundamental unit of HF information processing. Supporting homology between the avian and mammalian HF, context-specific patterns of Egr1 have been reported in birds that are comparable to those produced by place cell firing in mammals. Recent electrophysiological data, however, suggest that many avian species lack place cells, potentially undermining the correspondence between Egr1 and place cell-related firing in the avian brain. To clarify this, the current study examines Egr1 expression in Japanese quail under conditions known to elicit only weakly spatially modulated firing patterns and report robust context-dependent Egr1 expression. These data confirm that context-dependent expression of Egr1 is not dependent on precise place fields and provide insight into how these birds are able to perform complex spatial tasks despite lacking mammalian-like place cells.

## Introduction

The hippocampal formation (HF) is a structure critical to many forms of memory and spatial navigation in numerous species, including birds. For instance, the HF is required for learning maps of familiar landmarks (Gagliardo et al., 2001; White et al., 2002), locating baited food in a maze (e.g., Hough and Bingman, 2004; Ruploh et al., 2011; Damphousse et al., 2022), and the recovery of hidden food items (Sherry and Vaccarino, 1989).

One of the most striking features of mammalian HF is the ability for its cells to fire in a manner that reports an animal’s location in space (O’Keefe and Dostrovsky, 1971). Further observations have shown that these place cells respond to much more than merely spatial location—factors, such as task demands or internal state influence the firing of these cells (e.g., Markus et al., 1995; Skaggs and McNaughton, 1998; Wood et al., 2000; Kennedy and Shapiro, 2009), suggesting that place cell-like firing patterns form the fundamental units for the HF to record experience (reviewed by Eichenbaum, 2017; Epstein et al., 2017; Bellmund et al., 2018). Importantly, single-unit recordings have revealed some degree of spatially tuned firing patterns in a wide variety of animals, including several avian species (e.g., Hough and Bingman, 2004; Siegel et al., 2005; Agarwal et al., 2021; Ben-Yishay et al., 2021; Payne et al., 2021), suggesting that there may be widespread homology in this form of information processing across different classes of animal. Characterizing the homology of spatial information processing across the animal kingdom will require increasing the variety of animals in which place cell-like activity can be either confirmed or refuted.

Investigating the homology of spatial information processing may be aided by expanding the techniques available for assessing place cell-like activity. In mammals, the dynamics of place cell-related firing can also be examined through the expression of immediate-early genes, such as *Egr1*, which is tightly coupled to neuronal activity, a critical mediator of plasticity (Bozon et al., 2003), and reliably reports place cell activity (Marrone et al., 2011). Context-specific *Egr1* expression has been reported in hippocampal area DM in the brown-headed cowbird (Grella et al., 2016), suggesting place cell-like activity in these birds. Unfortunately, the spatial-tuning of cells in this species remains uncharacterized. This is problematic in that recent data show that both the abundance and location of spatially tuned cells in birds can vary substantially across species (Payne et al., 2021) and tasks (Kahn et al., 2008). Generating comparable data using both a species and behavioral conditions where the firing patterns are known will permit stronger conclusions to be made regarding the coupling between firing patterns and gene expression in the avian HF.

To accomplish this, the current experiment examines Japanese quail as they freely explore an open field, mimicking the conditions under which quail HF cells are weakly spatially coupled and are responsive largely to head direction (Ben-Yishay et al., 2021) in order to determine the extent to which these firing patterns can drive context-specific *Egr1* expression.

## Materials and Methods

### Subjects

Twenty-four Japanese quail (Spring Creek Quail Farms, St Ann’s, ON) were used in this experiment. All birds were group housed on a 12:12 h light cycle with *ad lib* access to food and water. Prior to behavioral testing, all birds were handled 15 min/day for at least 7 days. All procedures were approved by the animal care committee of Wilfrid Laurier University and were carried out in accordance with the guidelines of the Canadian Council on Animal Care.

### Behavioral Procedures

**Figure 1:**
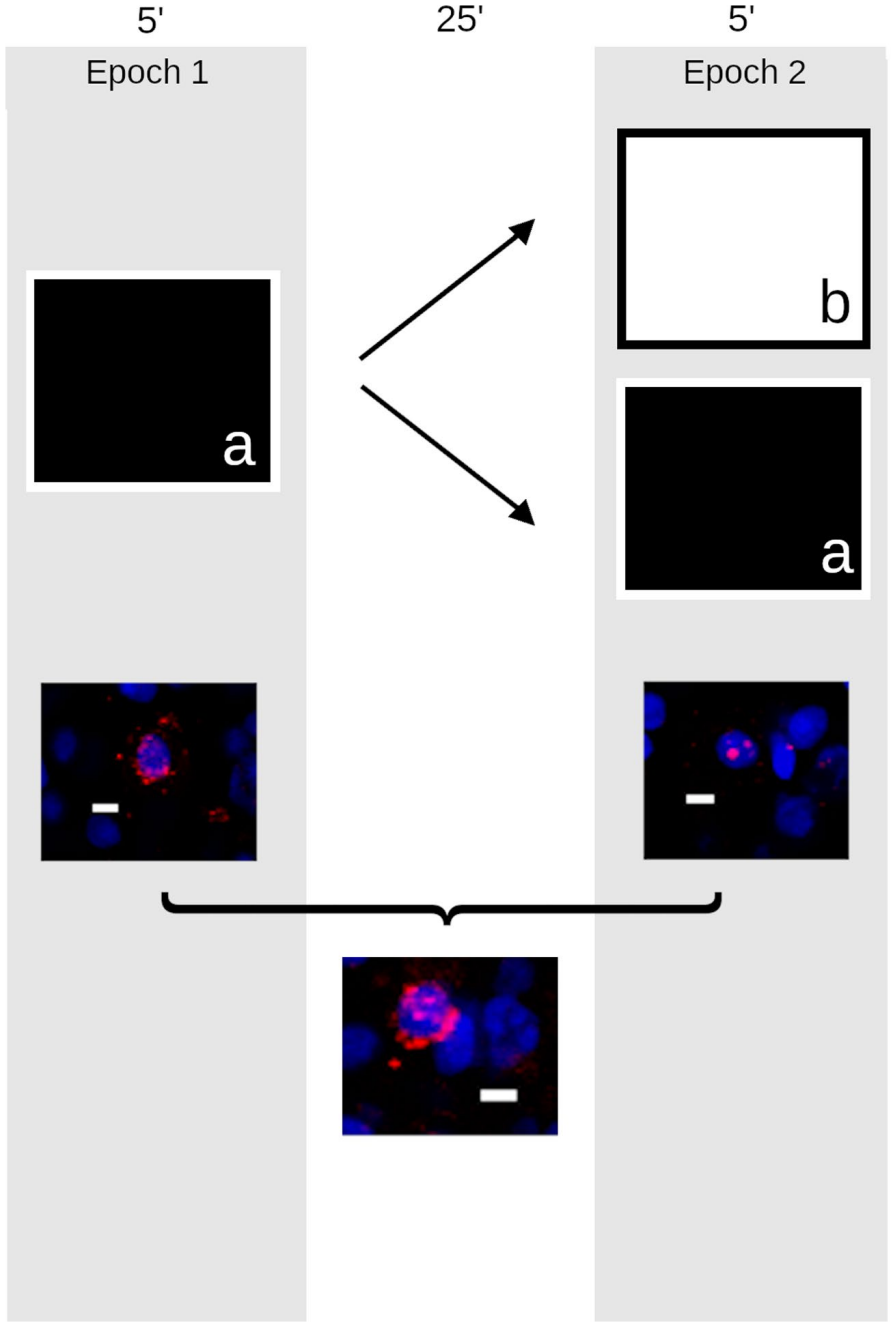
Schematic outline of the experiment. The timeline presented above shows that quail were taken to an open field (a) approximately 1 m^2^ containing distinct local and distal cues and provided passive exploration for 5 min (left). They returned to their housing room for 25 min before either being returned to the same environment (a) or placed in a new environment (b) in a different room containing a different complement of local and distal cues (right). Following these trials (below), the animals’ brains were harvested and the compartmental expression of *Egr1* (red) was measured to provide a histological record of activity in neurons counterstained with DAPI (blue) in the quail hippocampus (Scale bar = 10 μm). Cells active during the first epoch on the maze expressed *Egr1* within the cytoplasm (left); those active during the second epoch express *Egr1* within the nucleus (right). Cells active during both epochs express *Egr1* within both cellular compartments (bottom).

A modified version of the procedures outlined by Marrone et al. (2011) was used here (Figure 1). Briefly, two groups of quail were taken to a room containing several local and distal landmarks and passively moved through a 1 m^2^ open field. That is, the environment was divided into nine equally sized grids and the quail were moved to a new grid every 30 s for 5 min. Quail were then returned to their housing room. After a 25 min delay, quail were passively moved through either the same environment again (same, *n* = 8) or another open field in a second room containing distinct local and distal cues (different, *n* = 8). A third control group remained in the colony room undisturbed (caged, *n* = 8).

### Tissue Preparation

At the end of behavioral testing, quail were anesthetized by isoflurane and decapitated. Brains were removed and flash frozen in a container of 2-methyl butane submerged in a secondary container containing an ethanol and dry ice slurry. Brains were then mounted in optimal cutting temperature (OCT) medium (Fischer Scientific, Whitby, ON), sectioned (20 μm thick) on a cryostat, and thaw-mounted on Superfrost Plus slides (VWR Scientific).

Fluorescent *in situ* hybridization was then performed as previously described (Grella et al., 2016). Briefly, digoxigenin-labeled *Egr1* antisense riboprobes were synthesized using a transcription kit and digoxigenin-labeled UTP, denatured, and hybridized with the tissue overnight at 56°C. Following post-hybridization washes of gradually increasing stringency and 2% H_2_O_2_ to quench endogenous peroxidase, the riboprobe was detected with anti-digoxigenin-HRP and a CY3 tyramide signal amplification kit (Superglow red, Fluorescent Solutions, Augusta, GA). Slides were counterstained with DAPI, cover-slipped with anti-fade media, and sealed. All reagents are from Millipore Sigma (Oakville, ON) unless otherwise specified.

### Imaging

As described previously (Grella et al., 2016), *z*-stacks (optical thickness: 1.1 μm, interval: 0.7 μm) were obtained throughout the entire thickness (~20 μm) from area DM of the rostral hippocampus, approximately ~5.0–6.0 mm anterior to the interaural plane (Baylé et al., 1974) from 5 to 6 slides per animal. The lateral boundary of DM was defined by a shallow sulcus running rostrocaudally on the dorsal ventricular surface, and images were taken at the midway point from that boundary and the midline. Images were collected using an Olympus FV1000 laser scanning confocal microscope at 40× with consistent photomultiplier tube assignments, confocal aperture size, and contrast values for each slide. Image analysis was conducted using FIJI (Schindelin et al., 2012). Neurons were differentiated from glia on the basis of their size and uneven chromatin staining and were counted by the optical dissector within the median 20% of planes in each confocal stack and were classified as: (a) *Egr1*-negative, (b) *Egr1*-positive in the nucleus, (c) *Egr1*-positive in the surrounding cytoplasm, or (d) expressing *Egr1* in both the nucleus and cytoplasm (Figure 1). Because the localization of *Egr1* provides a marker of the time at which each neuron was engaged in transcription, gene expression profiles were used to calculate the number of cells active during spatial exploration, rest, or both time periods. Cell counts were conducted blind to the experimental group. As previously described (Grella et al., 2016), an overlap measurement was calculated as the percentage of cells containing Egr1 in both cellular compartments divided by the lesser of the number of cells active during epoch 1 and epoch 2.

Group differences in *Egr1* transcription were analyzed with a one-way ANOVA, with condition (i.e., same, different, and caged control) as the factor, followed by Tukey’s HSD *post-hoc* tests. In addition, the observed probability that cells express *Egr1* in response to both environmental exposures was compared to the probability expected by chance using a paired *t*-test. All statistical comparisons were completed using JASP (JASP Team, 2021).

## Results

### Equivalent Behaviorally Induced *Egr1* in the Left and Right Quail Hippocampus

No significant hemispheric differences were observed for Egr1 expression in either epoch 1 (*F*_1,42_ = 1.24; *p* = 0.27) or 2 (*F*_1,42_ = 2.80; *p* = 0.10), or in the overlap between the 2 (*F*_1,42_ = 2.01; *p* = 0.16). Moreover, no hemisphere by condition interactions were observed (*p* > 0.53 in all cases). These data indicate that this behavioral paradigm does not elicit a distinct response from the left and right hippocampus of quail.

### The Contexts Visited Alter the Pattern, but Not Number, of Cells Expressing *Egr1*

**Figure 2:**
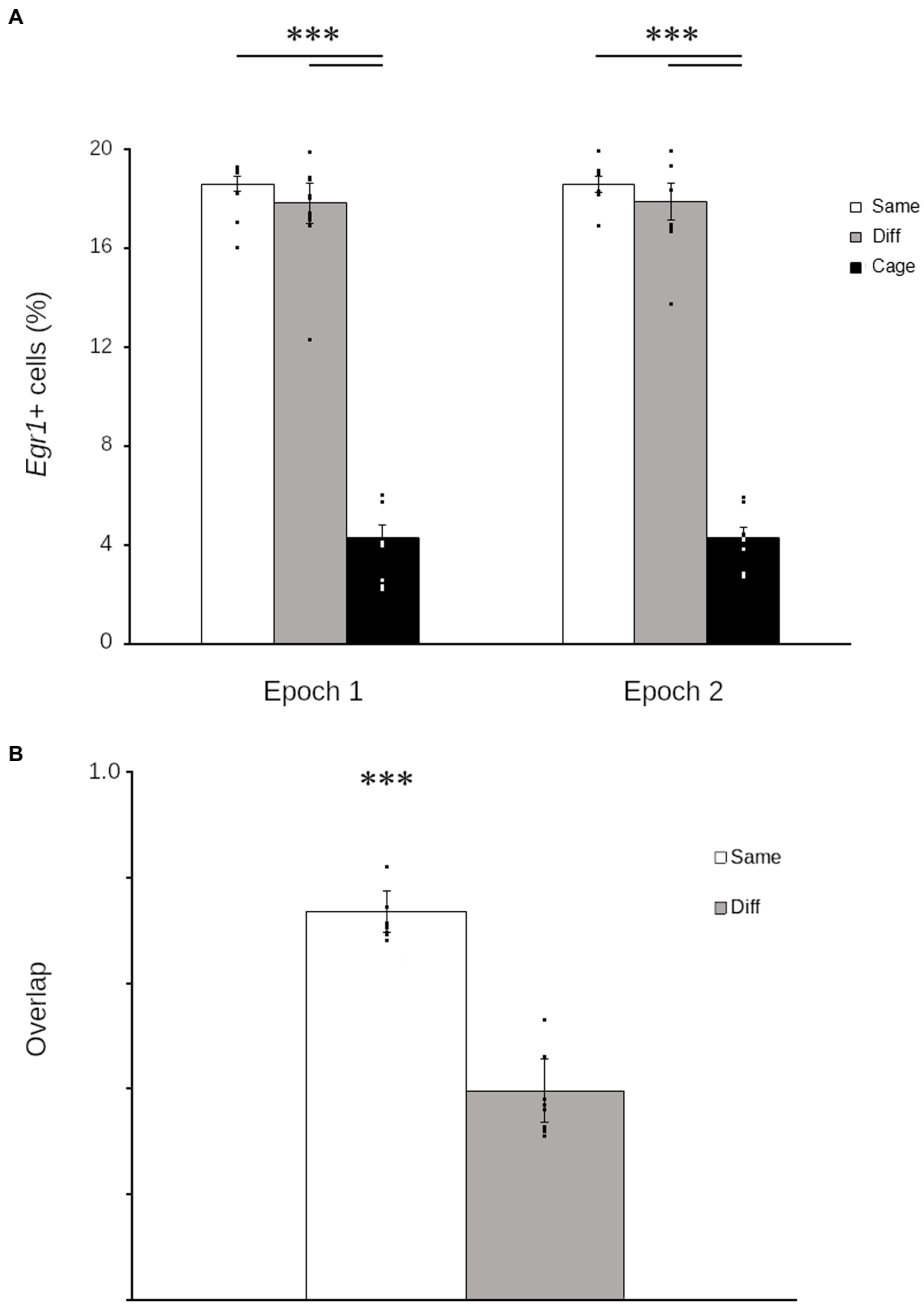
Hippocampal *Egr1* expression is context-specific in Japanese quail. Graphs of Egr1 expression during each epoch **(A)** show that quail that explored either the same environment *repeatedly* (white) or two different environments (grey) both expressed *Egr1* in significantly more cells than quail that remained in the housing room (black). Similarity scores **(B)** show that the proportion of cells that repeatedly expressed *Egr1* across both explorations was significantly higher in birds that explored the same room twice (Same: white) relative to birds that explored different rooms (Different: grey). Data are means ± SEM (^***^p < 0.001). Individual data points are for each quail are superimposed on each bar.

Exploration robustly increased *Egr1* in the quail HF both in epoch 1 (main effect: *F*_2,42_ = 241.94; *p* < 0.001) and 2 (main effect: *F*_2,42_ = 245.43; *p* < 0.001). In the HF of exploring quail, *Egr1* was consistently expressed in ~20% of cells (Figure 2A), somewhat higher than reported for cowbirds (Grella et al., 2016) but considerably lower than comparable estimates of cellular recruitment in rodents (e.g., Gheidi et al., 2012).

Despite the consistency in the number of cells transcribing Egr1, the number of environments explored significantly altered the pattern of *Egr1* expression (*t*_30_ = 14.07; *p* < 0.001). Quail that explored the same context twice demonstrated a significantly higher overlap relative to birds that explored two different contexts (Figure 2B). These data demonstrate that, as observed in mammals (e.g., Guzowski et al., 1999; Marrone et al., 2011) and other birds (Grella et al., 2016), the pattern of *Egr1* expression in the HF of quail is contextually mediated.

## Discussion

The current findings show that Japanese quail tested under conditions known to generate little place cell-like firing still recruit unique populations of cells in a context-specific manner. That is, when quail traverse through an environment, nearly 20% of cells transcribe *Egr1*, a proportion comparable, although marginally higher, than electrophysiological estimates under the same circumstances (Ben-Yishay et al., 2021). Moreover, the population of responsive neurons is fairly stable over time in the same location, as the vast majority of the cells are recruited again when quail return to the same environment. Importantly, a visually distinct environment results in the recruitment of a distinct population of cells, and the similarity of the recruited population of cells drops significantly when quail visit two distinct environments.

These results are remarkable in part because they expand our knowledge of the link between neural activity and immediate-early gene expression. Observing a context-specific pattern of *Egr1* under conditions that do not produce place cell-like firing demonstrates that place cell-like activity is not required to trigger context-specific transcription. Rather, the high frequency firing of either a head direction cell responding to its preferred heading or a place cell responding to its preferred location are equally capable of triggering plasticity-related gene expression cascade. Unfortunately, this observation limits the utility of activity-dependent gene expression as an *a priori* tool in the search for place cell-like patterns of activity in the avian hippocampus. The current data add to these observations by showing that the head direction system in quail is capable of recruiting a context-specific cohort of cells. Many questions remain to be answered about how this is accomplished, but even a rudimentary binding of head directions to the features of the stimuli present could accomplish this. The fact that the preferred direction of many head direction cells of quail rotate when cues are rotated (Ben-Yishay et al., 2021) supports this kind of binding. It is intriguing to note that while simple binding of visual information with head direction likely provides an effective basis for discrimination under many circumstances, it would be vulnerable to interference in the face of similar stimuli. This is consistent with the available behavioral evidence showing that Japanese quail perform poorly when discriminating multiple environments with shared features (Damphousse et al., 2021).

The observation that quail can generate context-specific patterns of activity despite having a hippocampal network composed largely of head direction cells may not be surprising, as head direction cells are thought to provide a potential neural basis of vector learning (Pearce et al., 1998; Kosaki et al., 2015). Moreover, the attractor dynamics in the head direction system are thought to confer mnemonic ability (Sharp et al., 2001). Thus, head direction cells in quail (and likely many other birds) may provide a navigational system that can support a wide variety of spatial computations. In fact, the Japanese quail’s ability to perform a number of complex spatial learning tasks is consistent with this idea. Quail show preference for previously blocked arms in a Y-maze (Damphousse et al., 2021), show conditioned place preference (Mace et al., 1997; Eaton et al., 2022), and complete foraging arrays comparable to a Barnes maze or Morris water maze (Ruploh et al., 2011; Damphousse et al., 2022).

It is important to note, however, that the observation that quail do not express large numbers of place fields under these conditions does not necessarily mean that they cannot express place fields. Previous observations in the pigeon establish that the spatial information in avian hippocampus is dramatically reduced in the absence of a spatial task with fixed reward locations (Kahn et al., 2008). In fact, a similar, although much less pronounced, phenomenon has been demonstrated in rodents, as the presence of a spatial task increases both the information content and stability of hippocampal firing patterns (Kentros et al., 2004).

If further observations support a lack of large numbers of place cells in Japanese quail, these animals will help us to define the role of place cells in spatial learning networks in which they are present in large numbers. In this way, Japanese quail may provide a strong model animal to test models of navigation without cognitive maps (e.g., Kubie and Fenton, 2009; Cruse and Wehner, 2011; Le Moel et al., 2019; Bouchekioua et al., 2021). These data promise to greatly clarify the unique contributions of the head direction system and place cells to spatial cognition.

## Data Availability Statement

The raw data supporting the conclusions of this article will be made available by the authors, without undue reservation.

## Ethics Statement

The animal study was reviewed and approved by Wilfrid Laurier University Animal Care Committee.

## Author Contributions

DM, NM, and CD designed the experiment and wrote the manuscript. CD and DM conducted the research and analyzed the data. All authors contributed to the article and approved the submitted version.

## Funding

This research was supported by the Natural Sciences and Engineering Research Council of Canada.

## Conflict of Interest

The authors declare that the research was conducted in the absence of any commercial or financial relationships that could be construed as a potential conflict of interest.

## Publisher’s Note

All claims expressed in this article are solely those of the authors and do not necessarily represent those of their affiliated organizations, or those of the publisher, the editors and the reviewers. Any product that may be evaluated in this article, or claim that may be made by its manufacturer, is not guaranteed or endorsed by the publisher.
